# Supplementary material for: The metabolome of male and female individuals with knee osteoarthritis is influenced by 18-months of weight loss intervention: the IDEA trial
Source: BMC Musculoskelet Disord. 2024 Dec 20;25:1057. doi: 10.1186/s12891-024-08166-7 (PMC11660641; doi:10.1186/s12891-024-08166-7)
Supplement: Supplementary file 1 — Supplementary Material 1 [file 12891_2024_8166_MOESM1_ESM.docx]

**Supplemental Materials – Methods, Results, Figures, Tables**

**Supplemental Methods**

Mass Spectrometry Instrumentation and Operation

The extracted serum samples (n = 90) were analyzed using liquid chromatography-mass spectrometry (LC-MS). Samples were analyzed in positive mode using an Agilent 1290 LC coupled through an electrospray ionization source to an Agilent Quadrupole Time of Flight (Q-TOF) mass spectrometer. Ions were separated using a Cogent Diamond Hydride HILIC chromatography column (2.2 µM, 120 Å, 150 mm x 2.1 mm, MicroSolv Leland, NC, United States) at a flow rate of 0.400 uL/min. For quality control purposes, the order of sample injection was randomized, 5 uL of each sample was injected, and blank samples containing neat 1:1 acetonitrile:water were injected every 10 samples.

Pooled samples were created by combining original extracts (n = 10). For each pool, 10 uL from 5 randomly selected participant samples were combined. This process was repeated for all pools (n = 10). Pooled samples were then subjected to liquid chromatography tandem mass spectrometry (LC-MS/MS) for metabolite identification purposes. Pooled samples were injected and analyzed using an Acquity UPLC Plus coupled through an electrospray ionization source to a Waters Synapt XS. Like serum samples, ions were separated using a Cogent Diamond Hydride HILIC chromatography column (2.2 µM, 120 Å, 150 mm x 2.1 mm, MicroSolv Leland, NC, United States) at a flow rate of 0.400 uL/min.

Pooled LC-MS/MS data were analyzed using Progenesis QI (Nonlinear Dynamics, Newcastle, UK, version 3.0). Data were imported, peaks were determined, and spectra were aligned. Next, acquired parent and daughter fragments were compared against theoretical fragmentation patterns using the Human Metabolome Database[56] for metabolite identification. We defined successful metabolite identifications as those with a Progenesis score greater than 60/100 and a fragmentation score > 20. The properties that contribute to these scores include mass error, isotope distribution similarity, and retention time error. Parts per million (ppm) error was calculated between LC-MS and LC-MS/MS data, and those with a ppm error greater than 20 were not considered.

**Supplemental Results**

Pairwise comparisons using delta change values between the three intervention groups were performed to further investigate how weight loss interventions differentially influence OA metabolism. PCA, PLS-DA, fold change, and volcano plot analyses were performed to investigate potential metabolic differences. When comparing DE and E groups, PCA analysis displays overlap, however, supervised PLS-DA shows distinct separation and very minimal overlap with 26.1% of the overall variability in the dataset being accounted for by the first two components (Supplemental Fig. 2A-B). Next, fold change analysis was conducted to detect populations of metabolite features most dysregulated between DE and E participants (FC > 2) Populations of metabolite features distinguished by fold change analyses were further examined to underpin differentially regulated metabolic pathways via MetaboAnalyst’s functional analysis program. Fold change analysis identified 760 metabolite features that had at least a 2-fold greater delta change among DE participants compared to E participants, mapping to pathways involved with glycosaminoglycan degradation, pantothenate and CoA biosynthesis, and terpenoid backbone biosynthesis. Conversely, 429 metabolite features had a greater delta change among E participants compared to DE, mapping to aminoacyl-tRNA biosynthesis, various amino acid metabolisms, glutathione metabolism, purine metabolism, and cytochrome P450 metabolism (Supplemental Fig. 2C, Supplemental Table 2).

The same analyses were used to compare DE and D participants. PCA shows some overlap of groups with PC1 and PC2 representing 36% of the variability in the dataset. (Supplemental Fig. 2D). PLS-DA shows a distinct separation of groups with no overlap (Supplemental Fig. 2E), further suggesting that changes in metabolism are distinct between DE and D interventions. Fold change analysis revealed 386 metabolites that had at least a 2-fold greater delta change in D participants than DE, mapping to pathways involved with aminoacyl-tRNA biosynthesis, cytochrome P450 metabolism, lysine degradation, tryptophan metabolism, purine metabolism, and glutathione metabolism. Comparatively, 650 metabolites had at least a 2-fold greater delta change in abundance during the 18-month period in DE participants than the D participants, mapping to terpenoid backbone biosynthesis, steroid hormone biosynthesis, N-glycan biosynthesis, and ubiquinone and other terpenoid-quinone biosynthesis (Supplemental Fig. 2F, Supplemental Table 2).

Finally, PCA comparison of D and E participants displays overlap between groups, with PCs accounting for 33.9% of the variability in the dataset (Supplemental Fig. 2G). PLS-DA shows complete separation of intervention groups (Supplemental Fig. 2H). Fold change analysis identified 340 features with a greater delta change (FC > 2) in the D group than the E participants but did not map to any significant pathways. Comparatively, 381 metabolite features had a greater change in abundance in the E group, mapping to N-glycan biosynthesis and ubiquinone and other terpenoid-quinone biosynthesis (Supplemental Fig. 2I, Supplemental Table 2). Considering these three pairwise comparisons between intervention groups, it is evident that intervention differentially influences the serum metabolome.

**Supplemental Figures**


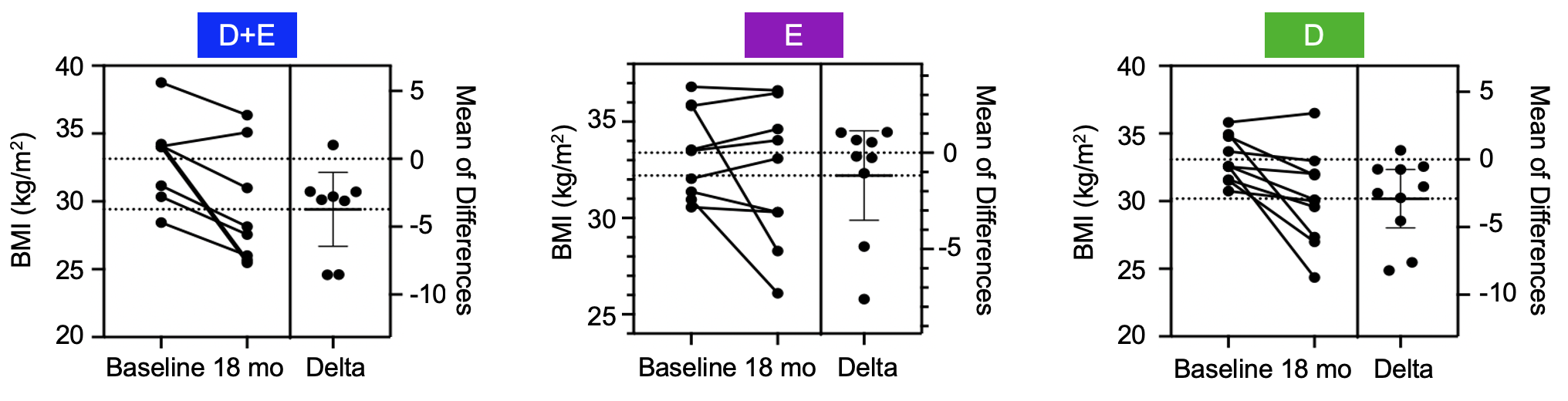


**Supplemental Fig. 1.** Line plots of changes in BMI (kg/m^2^) at baseline and after 18 months of intervention across D + E, E, and D participants. Colors correspond to: green – diet (D); purple – exercise (E); blue – diet and exercise (D + E).

**
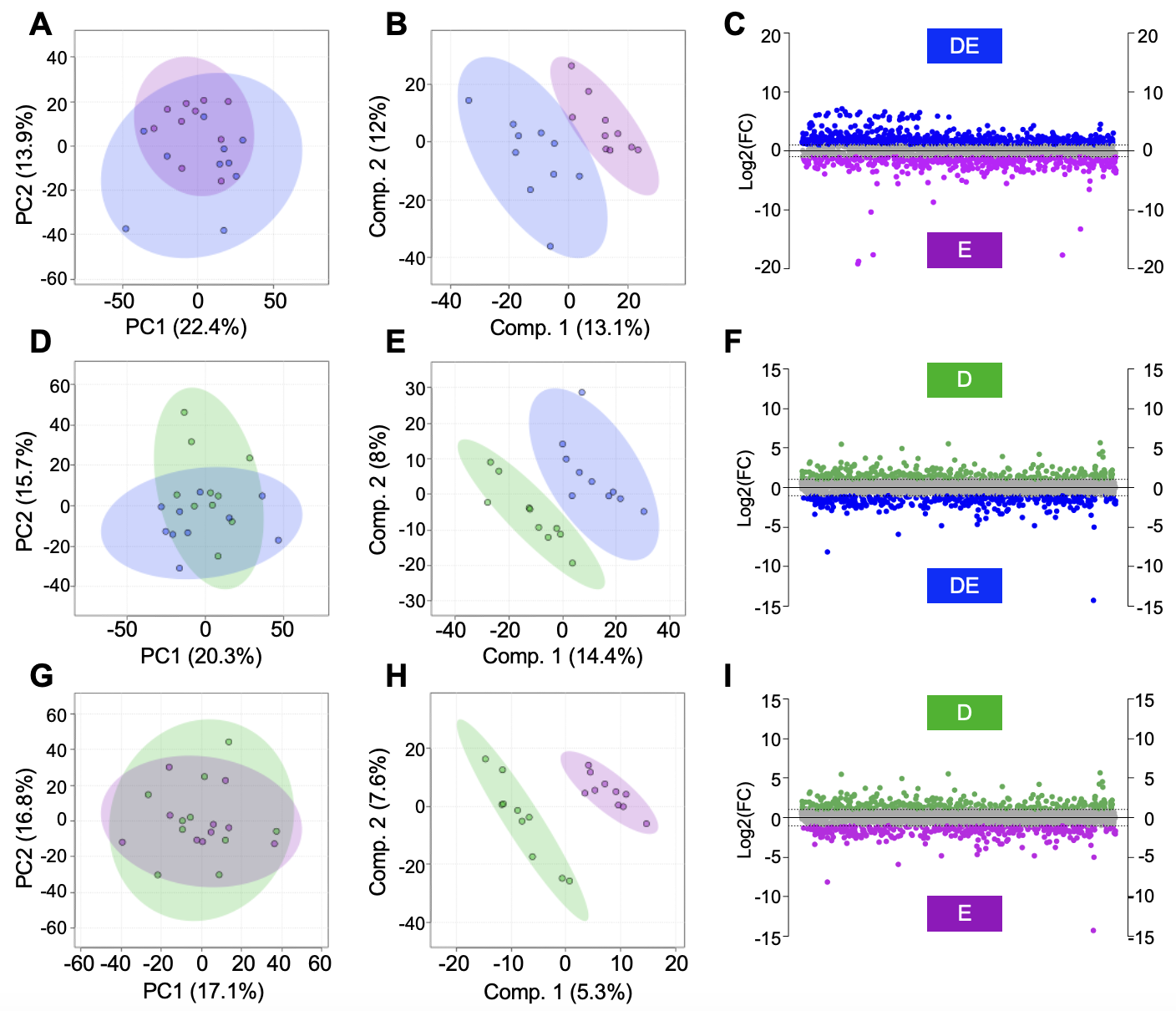
**

**Supplemental Fig. 2. Pairwise comparison unveil intervention-associated metabolic patterns.** (A) Principal component analysis and (B) partial least squares-discriminant analysis were applied to visualize the metabolome of DE and E participants after 18-months of intervention. (C) Fold change analysis identified 760 metabolite features that had a FC > 2 and were higher in abundance in DE participants. Conversely, 429 had a FC < -2 and were higher in abundance in E participants. Similarly, (D) PCA displayed some overlap, whereas (E) PLS-DA displayed clear separation of DE and D participants. (F) Fold change analysis was applied to examine metabolic differences associated with DE and D participants. This same suite of analyses was applied to investigate metabolic differences between E and D participants (G-I). Collectively, it is evident that the serum metabolome is influenced by intervention type, and differences in metabolic patterns reflect intervention status. The colors in A-I correspond to: green – diet (D); purple – exercise (E); blue – diet and exercise (DE).


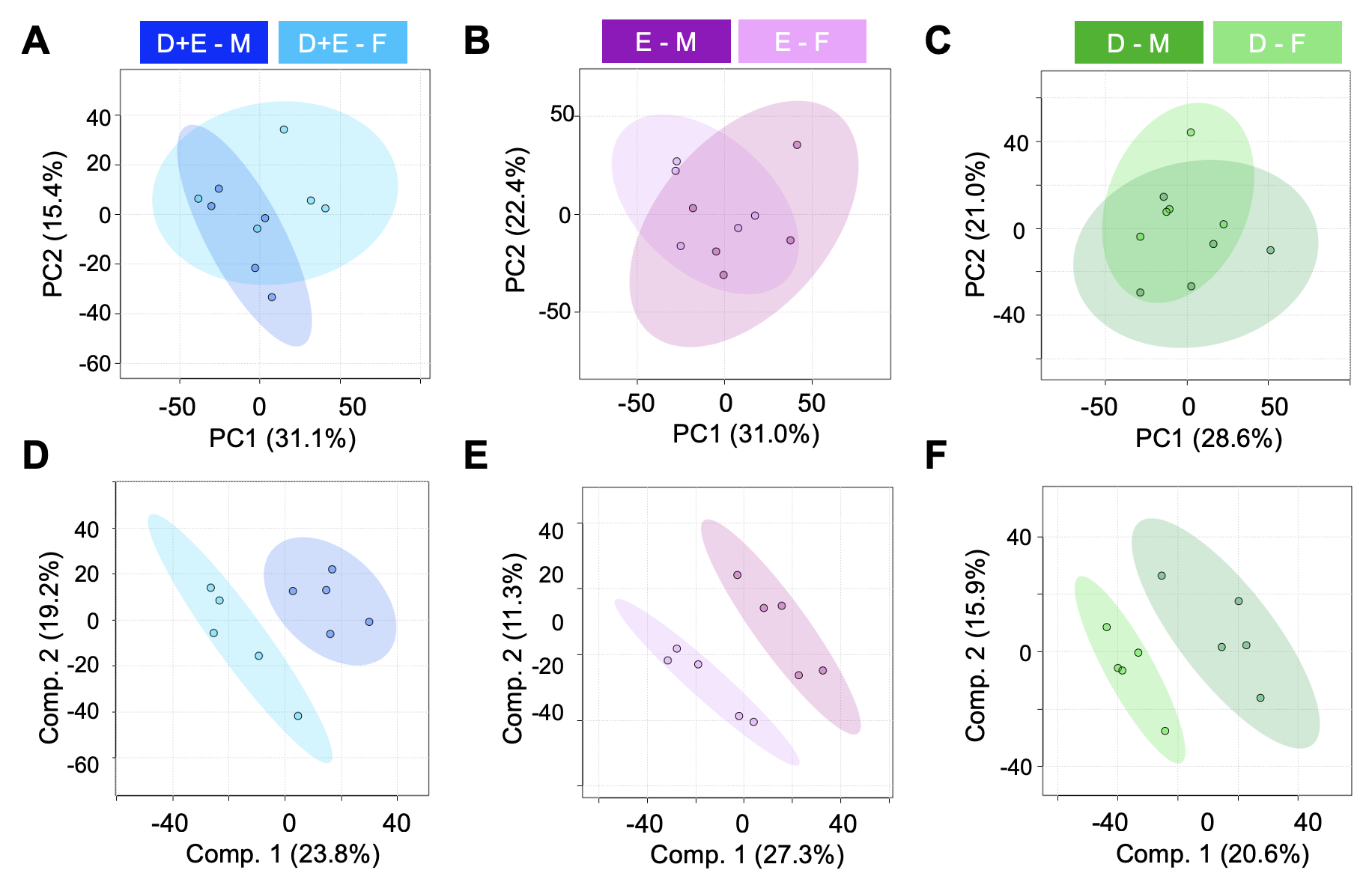


**Supplemental Fig. 3. Sex-associated metabolic patterns observed at baseline across interventions.** (A-C) Principal component analysis and (D-F) partial least squares-discriminant analysis were applied to display differences in the metabolome between sexes at baseline across each intervention. The colors in A-F correspond to: light and dark blue – female and male diet and exercise (D + E) participants; light and dark purple – female and male exercise (E) participants; light and dark green– female and male diet (D) participants.

**Supplemental Table Captions**

**Supplemental Table 1.** Participant information for all participants including assigned intervention, weight and BMI data at baseline, 6-months, and 18-months, as well as calculated percentage of weight lost and BMI reduction.

**Supplemental Table 2.** Metabolic pathways determined from MetaboAnalyst when comparing participants from different intervention groups using fold change analysis.

**Supplemental Table 3.** Overlapping pathways identified by both fold change analysis and median heatmap analysis.

**Supplemental Table 4.** Putatively identified metabolites that differ in abundance between IDEA participants considering intervention group and sex. Populations of metabolite features distinguished by fold change analyses investigating both factors were investigated and compared to identifications that were made by using LC-MS/MS. Numerous metabolites were distinguished in both fold change analyses investigating both intervention and sex, while a handful of metabolites were either intervention- or sex-associated only. For those identified, metabolite information includes observed and theoretical mass-to-charge ratios, parts per million (ppm) error, compound identifier from either chemspider or HMDB, compound description, chemical formula, total score out of 100, as well as fragmentation score. Metabolites with a ppm error greater than 20 ppm, total score < 60, and a fragmentation score < 20 were excluded from the analysis.

**Supplemental Table 5.** Metabolic pathways determined from MetaboAnalyst when comparing male and female participants within their respective intervention groups using fold change analysis.

**Supplemental Table 6.** Raw metabolomics data. Information provided for participants includes intervention group, sex, BMI, and timepoint (baseline, 6 months, 18 months).

[IMAGE-IDEA_BMCMSKDis_Tables.xlsx]
